# Free mate choice does not influence reproductive success in humans

**DOI:** 10.1038/s41598-017-10484-x

**Published:** 2017-08-31

**Authors:** Piotr Sorokowski, Agata Groyecka, Maciej Karwowski, Upma Manral, Amit Kumar, Agnieszka Niemczyk, Michalina Marczak, Michał Misiak, Agnieszka Sorokowska, Thomas Huanca, Esther Conde, Bogdan Wojciszke, Bogusław Pawłowski

**Affiliations:** 1 Institute of Psychology, University of Wrocław, Wroclaw, Poland; 2 Wildlife Institute of India, Dehradun, India; 3 Department of Psychotherapy and Psychosomatic Medicine, TU Dresden, Dresden Germany; 4 Centro Boliviano de Investigación y Desarrollo Socio-Integral, San Borja, Bolivia; 5 SWPS University of Social Sciences and Humanities, Faculty in Sopot, Sopot, Poland; 6 Department of Human Biology, University of Wroclaw, Wroclaw, Poland

## Abstract

The effect of free mate choice on the relative magnitude of fitness benefits has been examined among various species. The majority of the data show significant fitness benefits of mating with partners of an individual’s own choice, highlighting elevated behavioral compatibility between partners with free mate choice. Similarities between humans and other species that benefit from free mate choice led us to hypothesize that it also confers reproductive benefits in Homo sapiens. To test this hypothesis, we conducted a study among three indigenous societies—the Tsimane’, Yali, and Bhotiya—who employ natural birth control. In all three samples, we compared the marriages arranged by parents with the non-arranged ones in terms of number of offspring. Here, we show that there were no significant relationships between type of marriage and the total number of alive children and number of dead children among the three sampled groups. The presented study is the first to date to examine the fitness benefits of free mate choice in humans. In discussion we present limitations of our research and discuss the possibility of love having a beneficial influence in terms of the number of offspring.

## Introduction

The effects of free mate choice on the relative magnitude of fitness benefits have been examined among various species, including insects^1, 2^, mammals^3–5^, fish^6, 7^, and birds^8–10^. The majority of the data show significant fitness benefits of mating with partners of an individual’s own choice (but see: refs 2,7 and 8). Free mate choice might be adaptive in terms of number of offspring^2, 4, 9^, their growth rate^6^ and viability^1, 4, 9^, and offspring performance (e.g., in building nests)^3, 4^ or their probability of surviving infections^11^. Typically, studies have aimed to examine the fitness benefits of mating with self-chosen partners by comparing individuals who paired with a preferred partner with those given a non-preferred one^1, 3–6, 8, 11^ or a random partner from the population^9^ (see ref. 12).

In some of the aforementioned research, only the arranged group might include individuals who otherwise would never be chosen (i.e., low-quality individuals). To address this issue, Ihle *et al*.^12^ reported an experiment on zebra finch that controlled for variation in overall partner quality by comparing pairs of individuals who chose each other with pairs composed of random individuals chosen by another individual, finding that free mate choice pairs achieved 37% higher reproductive success than arranged pairs.

Authors have suggested that one reason for the beneficial effects of free choice mating might be that these pairs are more compatible than arranged pairs. In the latter case, both sexes invested less in reproduction (i.e., less positive response to within-pair courtship and thus less frequent copulatory behaviors) and showed poorer coordination in protecting offspring^12, 13^. Another factor contributing to reproductive success is the similarity between individuals’ behavioral traits (e.g., in zebra finches)^14^. Thus, between-mate behavioral compatibility may be evolutionarily significant in birds. Meanwhile, several authors have ascribed fitness benefits to the genetic rather than behavioral compatibility of freely chosen mate pairs^1, 3, 4, 11^. They suggested that the best mate for any particular female/male may not be the best mate for another: in the context of immunorelated genes mate preferences may enhance offspring viability by disassortative mating. For example, such genetic similarity has been linked to elevated incidence of fetal loss^15, 16^, longer inter-birth intervals^15^ and lower resistance to evolving pathogens^3^. Moreover, studies have shown that allele sharing within social pairs of several bird species predict levels of extra-pair paternity and copulations (e.g., refs 17 and 18).

Ihle *et al*.^12^ (and other authors commenting on his work^19, 20^) suggested that because humans mate for life (or long periods), and the male plays a significant role in raising offspring, *Homo sapiens* should also show the fitness benefits of non-arranged mating. Ethical constraints prevent us from conducting similar human experiments to those done on birds. However, a comparison between marriages arranged by parents and those based on the spouses’ independent decisions could serve as an ethically admissible equivalent of manipulations of non-human animal mating. Although there is evidence that couples are more satisfied, engaged, and less prone to domestic violence within love-based than arranged marriages^21, 22^, research results in this area have been inconsistent (see ref. 23).

In the present study, we tested the hypothesis that free mate choice confers a fitness benefit on humans. We compared arranged marriages with free-choice marriages from cultures with natural fertility. The study was conducted on three different traditional societies of *Homo sapiens* with natural birth control: the Tsimane’ (Amazonia), Yali (West Papua), and Bhotiya (Himalayas). Its aim was to establish the relationship between type of marriage and number of offspring. To the authors’ knowledge, this is the first study to examine the reproductive benefits of free-choice mating in humans. The studied societies are culturally diversified and inhabit different parts of the globe. Below, we present a short description of each society; however, our intention was not to compare these societies but to test whether the hypothesis finds support in diverse cultures.

## Material and Methods

The study was conducted among three indigenous societies, the Tsimane’, Yali, and Bhotiya people; details on the three groups are presented in the *Participants* section. In all societies, we collected data on the number of children and relationship type (arranged vs. free-choice). In each society, we controlled for participants’ wealth—details on this measure within each group are described in the *Participants* section. The data were collected during individual interviews conducted by the authors with the help of a local interpreter. All data used in this paper were originally collected for the purpose of other studies, but they enabled us to analyze the differences in fertility and mortality between arranged and free-choice marriages.

### Ethical approval of the study protocol

The study was conducted according to the principles expressed in the Declaration of Helsinki. The study protocol and consent procedure received ethical approval from the Institutional Review Board (IRB) of the University of Wroclaw (Wrocław, Poland), the Great Tsimane’ Council (the governing body of the Tsimane’), and the head of the local Yali community in West Papua. Conducting studies among the Bhotiya is not restricted by any governmental regulations. The study has been conducted with respect to guidelines listed by Indian Council of Medical Research described by Sajitha and Ramanathan^24^. The approval from IRB regarded all three populations. Participants provided informed consent before study inclusion. The participants’ low literacy levels led us to obtain oral consent for participation.

### Participants

#### The Bhotiya

The Bhotiya (or Bot) are ethnolinguistically related to the Tibetan people and live in the Himalayas. Our study was conducted among the *Tolcha* and *Marcha* Bhotiya subgroups of Uttarakhand, Western Himalaya, an ethnic community of Indo-Mongoloid origin. They reside in the Niti valley of the Nanda Devi Biosphere Reserve in Uttarakhand, India. The upper catchment of the Niti valley has seven migratory villages (Kailashpur, Mahergaon, Gurgutti, Farkia, Bampa, Gamsali, and Niti), each consisting of two permanent settlements: one winter settlement at lower elevation in the Chamoli district and one at higher elevation (i.e., the summer settlement, Niti valley). The total number of households in the valley is 292 (total population: 864 people; Census India, 2011). The Bhotiya are monogamous, and marriages are mostly arranged by parents^25^, but free-choice pairs are also common. We collected the data in 2015. For more information about this group, see refs 26 and 27.

In the Bhotiya society, we collected data for 52 total couples, of which 31 and 21 were arranged and free-choice marriages, respectively. We only included those couples in which the answers of a husband and a wife regarding their marriage were consistent. The couples had an average of 2.31 (*SD* = 1.25) children and had been together for an average of 10.6 (*SD* = 6.3) years. We collected all data for both partners in a relationship. The participants’ wealth was defined as the value of the familial belongings (i.e., land size, livestock numbers) possessed by household members.

#### The Tsimane’

The Tsimane’ are a native Amazonian society of farmer-foragers. Their population of around 8,000 is distributed throughout approximately 100 villages, most of which are located in the Beni area of northern Bolivia. The studies among the Tsimane’ were conducted in several villages in the Maniqui river region (i.e., Campo Bello, Las Palmas, Las Minas, Uachichi, Maracas, Catumare, and Anachere) in 2012–2015. The Tsimane’ are a native Amazonian group, but their level of integration to the Bolivian economy, culture, and lifestyle varies between their settlements. This tribe has been extensively described in the literature (e.g. refs 28–30). Similar to other native Amazonian societies, the Tsimane’ still practice cross-cousin marriage. Traditionally, marriage is arranged by parents^31^, but currently, many couples claim to have been established without parental influence.

The Tsimane’ sample comprised 160 couples, of which 63 and 97 were arranged and free-choice marriages, respectively. We only included couples in which the answers of the husband and a wife regarding their marriage were consistent. The couples had an average of 4.64 (*SD* = 3.0) children and had been together for an average of 11.6 (*SD* = 9.5) years. We collected all data for both partners in a relationship. The participants’ wealth was defined as the value of cattle and estates possessed by household members.

#### The Yali

The Yali are a native Papuan (Indonesian) tribe that inhabits Yalimo, a mountainous terrain east of the Baliem valley. For extensive description of the tribe, see refs 32 and 33. The Yali have only minor contact with Western culture because of the difficult-to-access, remote location of their dwellings^32^. The Yali are polygamous, and their sexual life is limited by certain traditional restrictions, both before and after marriage. When a woman finds out that she is pregnant, the husband ceases to sleep in the family hut, and sexual intercourse is prohibited for about 4 years after birth (i.e., until the child is relatively independent^33^). However, some people may not comply with these rules^32^. Both types of marriages—free-choice and arranged—are common among the Yali.

The Yali sample comprised 101 married individuals, among whom 44 and 57 were in arranged and free-choice marriages, respectively. We collected the data in 2016. The couples had an average of 3.33 (*SD* = 2.9) children. The participants’ wealth was defined as the number of pigs possessed by household members.

## Results

All participants’ descriptive statistics are summarized in Table 1. To examine whether free mate choice affected reproductive success among humans, we conducted a series of regression analyses. Specifically, in the first regression model, we regressed the pair’s number of living children onto a dichotomously coded variable with 0 = free choice and 1 = arranged marriage. Additionally, we controlled our estimates for participants’ age, wealth, and possibly variable effects of arrangement across the three sites. Thus, we introduced two dummy variables: Tsimane’ (0 = no, 1 = yes) and Yali (0 = no, 1 = yes) and two cross-product terms: Tsimane’ × Arranged and Yali × Arranged. Coefficients of dummy variables (Tsimane’ and Yali) should therefore be read as the difference in the dependent variable between each of these groups and the Bhotiya, the reference category. Similarly, the interaction terms show whether the effect of free choice versus arranged marriage was significantly different in each of these two populations as compared with the Bhotiya. In the second regression model, the same procedure was conducted on the number of dead children (log-transformed because of skewness) as the dependent variable. We decided to proceed with multiple regression analysis on log-transformed dependent variables to facilitate interpretability. It should be noted, however, that Poisson regression with the raw number of dead children regressed onto the set of our predictors demonstrated exactly the same results. All analyses were realized using the Hayes^34^ macro process with 10,000 bootstrap samples and yielded unstandardized coefficients.

**Table 1:** Descriptive statistics for main variables used in the study.

Total (*N* = 313)	Min	Max	M	SD
Children	0	12	3.83	2.71
Children-dead	0	7	0.59	0.99
Children-dead (log transformed)	0	2.08	0.33	0.47
Arranged (0 = no, 1 = yes)	0	1	0.44	0.50
Age	15	80	34.89	13.55
Wealth*	−1.43	4.89	0	1
**Bhotiya (** ***N*** = **52)**				
Children	0	5	2.31	1.25
Children-dead	0	2	0.25	0.56
Children-dead (log transformed)	0	1.1	0.16	0.33
Arranged (0 = no, 1 = yes)	0	1	0.60	0.50
Age	18	40	29.39	5.24
Wealth	−0.70	4.37	0	1
**Tsimane’ (** ***N*** = **160)**				
Children	0	12	4.64	3
Children-dead	0	3	0.56	0.84
Children-dead (log transformed)	0	1.39	0.33	0.46
Arranged (0 = no, 1 = yes)	0	1	0.39	0.49
Age	15	80	32.68	12.86
Wealth	−1.43	4.89	0	1
**Yali (** ***N*** = **101)**				
Children	0	12	3.33	2.29
Children-dead	0	7	0.80	1.30
Children-dead (log transformed)	0	2.08	0.42	0.53
Arranged (0 = no, 1 = yes)	0	1	0.44	0.50
Age	18	75	41.24	15.21
Wealth	−1.08	4.24	0	1

*Wealth index was calculated for each population separately and standardized within each population.

The prevalence of free choice versus arranged marriage was not related to number of living children (*p* = 0.39), and number of dead children (*p* = 0.99). Importantly, this null effect was replicated across the three studied societies (see Table 2 for the summary of the regression analyses). No interactions for different effects of free choice across cultures were significant in any case, showing the overall stability of the null hypothesis across cultures. Not surprisingly, age and wealth were positive predictors of the number of living children. We also observed that when we controlled for age and wealth, the Tsimane’ had more children on average than the Bhotiya or Yali had.

**Table 2:** A summary of moderated-regression analyses with free-choice-versus-arrangement marriage and control variables explaining the number of living children, and the number of dead children.

	Alive Children			Dead Children (log-transformed)		
	*B* (*SE*)	95% *CI*	*p*	*B* (*SE*)	95% *CI*	*P*
Constant	2.19 (0.46)	1.30–3.09	<0.001	0.24 (0.09)	0.05–0.43	0.01
Arranged (0 = *no*, 1 = *yes*)	0.51 (0.59)	−0.65–1.66	0.39	−0.001 (0.12)	−0.24–0.24	0.99
Age	1.08 (0.13)	0.82–1.33	<0.001	0.20 (0.03)	0.15–0.25	<0.001
Wealth	0.39 (0.12)	0.15–0.63	0.0003	0.04 (0.03)	−0.02–0.08	0.17
Tsimane’	2.03 (0.50)	1.05–3.01	<0.001	0.13 (0.10)	−0.08–0.33	0.23
Yali	−0.49 (0.55)	−1.58 - 0.59	0.37	0.08 (0.11)	−0.14–0.31	0.46
Arranged × Tsimane’	−0.41 (0.68)	−1.74–0.92	0.54	−0.01 (0.14)	−0.29–0.26	0.94
Arranged × Yali	0.23 (0.72)	−1.19–1.65	0.75	0.01 (0.07)	−0.28–0.31	0.92
R^2^	0.33			0.21		

Note B = unstandardized regression coefficient. SE = standard error of B; CI = 95% confidence intervals; R2 – the percentage of dependent variable’s variance explained.

**Figure 1:**
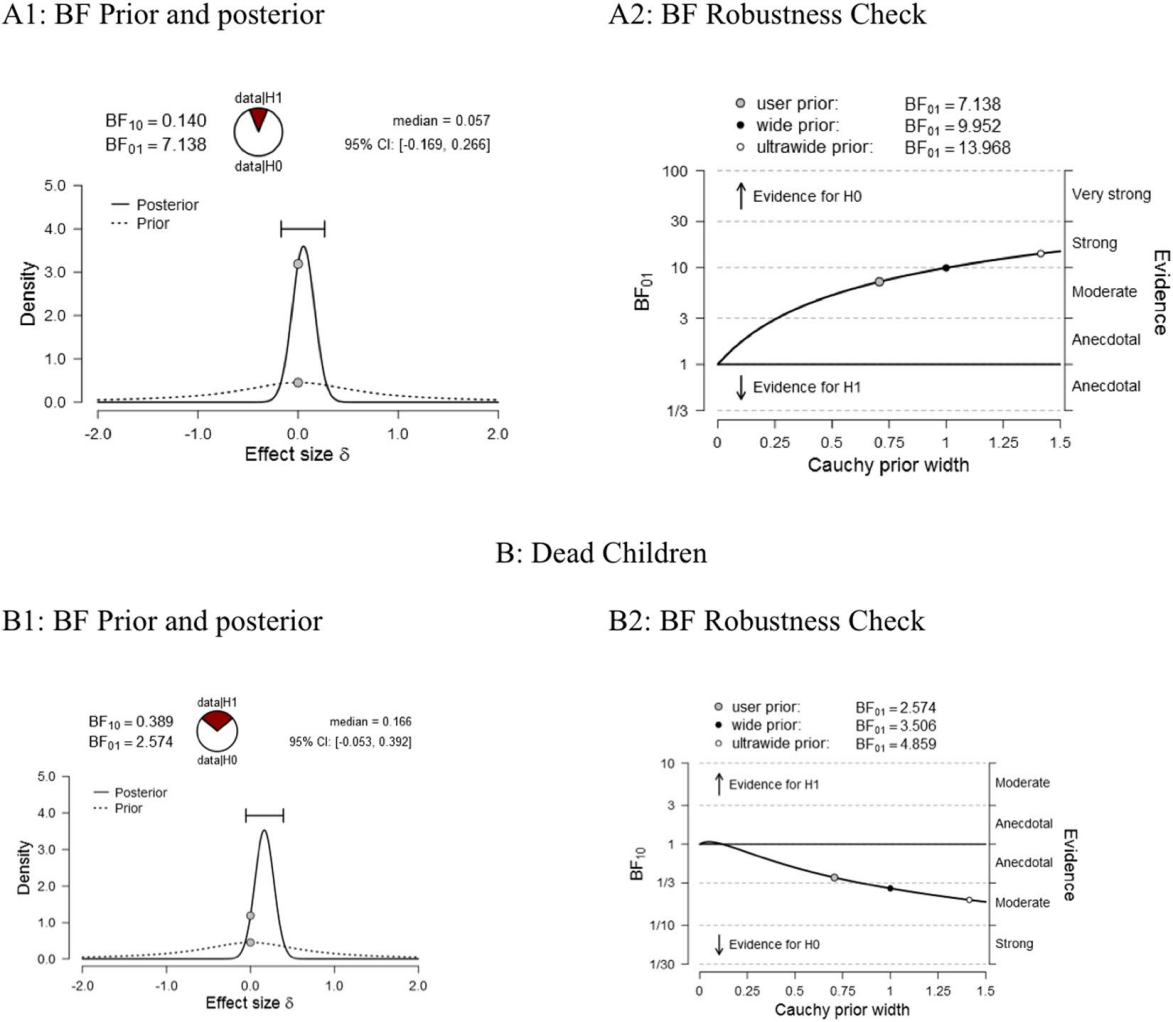
The Summary of Bayesian Analyses Results. Panels A1, and B1 present prior’s and posterior’s distribution in Bayesian Analysis as well as provide the values of Bayes Factors: BF_10_: providing the relative evidence for alternative hypothesis and BF_01_: providing the relative evidence for null hypothesis. In the case of the number of living children (panel A1), and the number of dead children (panel B1) the probability that the null hypothesis is more plausible based on data at hand is several times higher than that the probability that the alternative hypothesis is true. Panels A2, and B2 present Bayes Factor Robustness Check analysis, showing how the effect changes depending on the Cauchy prior probability. In the case of the number living of children (panel A2), the number of dead children (panel B2) the evidence for the null hypothesis under the default prior (*d* = 0.707) is moderate. Bayes factor analyses were conducted in JASP statistical software^60^.

The pattern observed in the regression models was consistent: in no case did free choice increase the number of children or the likelihood of their survival over arranged marriage. These results indicate that the null hypothesis should not be refuted. The results tell us little, however, about whether we should consider the null hypothesis as more plausible than the alternative hypothesis. To estimate the likelihood that the null versus directional hypothesis finds stronger support in our dataset, we proceeded with a series of Bayesian analyses. Specifically, using a series of Bayesian ANOVAs and independent-samples *t*-tests, we estimated the Bayes factors and conducted a robustness check (see Fig. 1).

In all analyzed cases, there is clear support for the null hypothesis. When the total number of living children is considered, BF01 = 7.14; therefore, the null hypothesis should be considered as 7 times more probable than the alternative one—a moderately strong pattern. Analysis of the number of dead children revealed that the null hypothesis is 2.5 times more likely than the alternative (i.e., anecdotal–moderate strength of evidence).

For some individuals in the Tsimane’ sample (*N* = 107), we had data on the time they had their first child after getting married (min = 0, max = 16 years, *M* = 2.21, *SD* = 2.77), but we observed no significant differences between arranged pairs (*N* = 51, *M* = 2.25, *SD* = 2.60) and free mate choice (*N* = 56, *M* = 2.17, *SD* = 2.93): *t*(105) = −0.15, *p* = 0.88, BF01 = 4.84. The Bayes factor indicates that the null hypothesis is almost 5 times more likely than the alternative in this case.

## Discussion

In contrast to data on many non-human animals^1, 3–6, 9, 11^, our results on free mate choice in humans from three different groups of indigenous people indicate that non-arranged marriages have no beneficial influence on reproductive success. Free-choice couples in Tsimane’, Yali, and Bhotiya tribes did not have more children or lower child mortality rates than arranged marriages. Below, we discuss the possible causes of such a result among humans.

Among non-human animals, the non-arranged and arranged pairs differed in reproductive investment: for example, they copulated less frequently and showed poorer nest attendance^12^, resulting in lower viability of their offspring. Free choice mating also influenced their number of offspring^4, 9^. One possible reason for the lack of this effect among humans is that humans have fewer offspring than other species^35^, and the average number of children born per human pregnancy is much lower (e.g., the number of eggs per clutch in zebra finch ranges 2–7, with 5 being most common^36^), possibly resulting in more cautious care for children among humans. The overall mortality rate in humans is also lower than that in non-human animals (0.31% in humans in Bolivia^35^ compared with up to 50% in rabbits^37^), and their viability is dependent not only on parental investment, parental care, or care of the closest environment but also on broader social context (e.g., grandparents). Besides this, we cannot exclude the possibility that caring behaviors are more specific for humans than non-human animals.

Although researchers investigating the evolutionary repercussions of non-human animals’ forced mating have been limited to experimenter-dependent or random mates, human studies have considered parental influence on mating with this regard. Empirical research has identified that good looks (as a proxy of genetic quality) and exciting personality are preferred more in a spouse than in an in-law, while priorities concerning in-laws include religiousness and family background^38–41^. Regardless of this conflict, traits that parents expect their sons- or daughters-in-law to have seem beneficial to them and their kin (i.e., good character, good family background, industriousness^42^) because they are benefited by grandchildren with good chances of survival and reproduction. Therefore, children and their parents may often agree in their mate preferences. This accordance might also be a result of the heritability of preferences for multiple cues, which is higher in females than males^43^. In addition, parental preferences, although not always identical with those of their children, may increase the frequency of certain traits in a population, as traits that are appealing to other men as sons-in-law confer reproductive benefits^44^. The fact that some traits become more widespread over society can result in their more favorable perception as people become more familiar with them. Also, parental decisions may also be influenced by children (although in situations where marriages are strictly arranged, as in the case of the Tiwi of Australia, where females are married off at birth^45^, this influence cannot occur). In summary, as stated in the introduction, comparisons between marriages arranged by parents and those based on the spouses’ independent decisions are the only ethically admissible equivalent of manipulations of animal long-term mating. However, it needs to be noted that arranged marriages do not have to mean no choice at all on the side of the children, and free-choice marriages might also be influenced by parents. Further, even though people in arranged marriages live with the partners chosen by their parents, they may have sex and reproduce with others, probably without their partners’ awareness.

Alternatively, considering their children’s future well-being, parents may try to match them with partners that are behaviorally alike; this is a key component of behavioral compatibility both in humans and other monogamous species^46, 47^. However, even if arranged couples differ in behavioral style at the beginning of their marriage, their within-couple similarity may increase after time spent together^48–51^. As has been shown in a monogamous fish species, mismatched partners that achieve post-pairing behavioral similarity improve their reproductive success^52^. Thus, *Homo sapiens* partners may also try to adjust their behavior to be more like their partners to adapt to unfavorable situations. However, in the growing scope of literature on within-marital similarities in humans, it is still not certain whether the similarities between partners are a matter of convergence or selection, and much research has failed to support the convergence hypothesis^50, 53^. Other studies suggest that the strength of assortative mating varies within couples as for some traits this effect is much stronger than for others^54^.

We might also present other hypothesis. Assuming that non-arranged marriages are based on love, one may conclude that love does not necessarily favor humans’ fitness in traditional societies. These kinds of conclusions, however, require caution and precise definitions, as love is a complex psychological construct and probably not all of its components play roles in the same stages of the relationship^55^. There are numerous, not mutually exclusive, typologies of love. For example, Sternberg’s Triangle Theory of Love suggests existence of three components – passion, intimacy and commitment^56^. Alternatively, according to Hatfield and Rapson’s model, one can distinguish between two types of love – passionate and companionate^57^. In both approaches, love is dynamic and changes in time. Passion is the strongest element on the early stages of relationship formation, while other factors related to attachment (and a will to reproduce) may appear later, likely also in arranged marriages (especially that arranged marriages and marriages of choice do not differ in marital satisfaction^58^). Therefore, our findings might be only conclusive for passionate love, which is considered to be the dominant element of in the beginning of free-choice relationships^56^.

Finally, the inconsistency between the results among non-human animals and humans may be caused by differences in study design, which for ethical reasons could not be identical. While non-human animals were forced to mate with other individuals after choosing (i.e., showing interest in) their preferable mates, humans ‘forced’ to mate by means of arranged marriage did not make a prior choice. They were probably aware that their spouses’ identity would be a parental decision, and this might have resulted in lower engagement with the mating process. Thus, some of them might have avoided developing trauma caused by separation from their beloved, as occurs in non-human animal experiments (e.g., ref.^12^).

Also, in contrast to some research on other species (e.g., ref. 1), the arranged marriages investigated in our study design did not consist of partners with noticeably lower quality, who would have otherwise not mated at all, as mates were selected by the parents. It needs to be noted again that the fact that an individual was forced to marry someone does not necessarily imply that the individual would be rejected given free choice.

Moreover, our results should not serve as devaluation of previously well documented benefits following successful pair bonding. Well-matched pairs are described to experience better physical and mental health and they have a significant advantage in raising children in comparison to individuals who decided to end their relationship^59^.

Despite these unavoidable limitations, the presented study is the first step to understanding the relationship between arranged mating and humans’ reproductive success. Although the aforementioned factors may have influenced the quality of arranged marriages, none of them can exclusively explain the obtained results, and therefore, future research in this field is required, preferably with control for the degree to which future spouses were satisfied with their parents’ decision.
