# Author Correction: Free mate choice does not influence reproductive success in humans

**DOI:** 10.1038/s41598-018-28336-7

**Published:** 2018-07-03

**Authors:** Piotr Sorokowski, Agata Groyecka, Maciej Karwowski, Upma Manral, Amit Kumar, Agnieszka Niemczyk, Michalina Marczak, Micha Misiak, Agnieszka Sorokowska, Thomas Huanca, Esther Conde, Bogdan Wojciszke, Bogusław Pawłowski

**Affiliations:** 1 Institute of Psychology, University of Wrocław, Wroclaw, Poland; 2 Wildlife Institute of India, Dehradun, India; 3 Department of Psychotherapy and Psychosomatic Medicine, TU Dresden, Dresden, Germany; 4 Centro Boliviano de Investigación y Desarrollo Socio-Integral, San Borja, Bolivia; 5 SWPS University of Social Sciences and Humanities, Faculty in Sopot, Sopot, Poland; 6 Department of Human Biology, University of Wroclaw, Wroclaw, Poland

Correction to: *Scientific Reports* 10.1038/s41598-017-10484-x, published online 31 August 2017

The Acknowledgements section in this Article is incomplete.

“We thank D.M. Buss for his comments.”

should read:

“We thank D.M. Buss for his comments. Study was funded by National Science Centre-PL-2014/13/B/HS6/02644”

